# 3D renal model for surgical planning of partial nephrectomy: A way to improve surgical outcomes

**DOI:** 10.3389/fonc.2022.1046505

**Published:** 2022-10-21

**Authors:** Lorenzo Bianchi, Laura Cercenelli, Barbara Bortolani, Pietro Piazza, Matteo Droghetti, Sara Boschi, Caterina Gaudiano, Giulia Carpani, Francesco Chessa, Simone Lodi, Lorenzo Tartarini, Alessandro Bertaccini, Rita Golfieri, Emanuela Marcelli, Riccardo Schiavina, Eugenio Brunocilla

**Affiliations:** ^1^ Division of Urology, IRCCS Azienda Ospedaliero-Universitaria di Bologna, Bologna, Italy; ^2^ Università degli studi di Bologna, Bologna, Italy; ^3^ eDIMES Lab - Laboratory of Bioengineering, Department of Experimental, Diagnostic and Specialty Medicine (DIMES), University of Bologna, Bologna, Italy; ^4^ Department of Radiology, IRCCS Azienda Ospedaliero-Universitaria di Bologna, Bologna, Italy

**Keywords:** 3D model, surgical planning, surgical outcomes, renal cancer, partial nephrectomy

## Abstract

**Objective:**

to evaluate the impact of 3D model for a comprehensive assessment of surgical planning and quality of partial nephrectomy (PN).

**Materials and methods:**

195 patients with cT1-T2 renal mass scheduled for PN were enrolled in two groups: Study Group (n= 100), including patients referred to PN with revision of both 2D computed tomography (CT) imaging and 3D model; Control group (n= 95), including patients referred to PN with revision of 2D CT imaging. Overall, 20 individuals were switched to radical nephrectomy (RN). The primary outcome was the impact of 3D models-based surgical planning on Trifecta achievement (defined as the contemporary absence of positive surgical margin, major complications and ≤30% postoperative eGFR reduction). The secondary outcome was the impact of 3D models on surgical planning of PN. Multivariate logistic regressions were used to identify predictors of selective clamping and Trifecta’s achievement in patients treated with PN (n=175).

**Results:**

Overall, 73 (80.2%) patients in Study group and 53 (63.1%) patients in Control group achieved the Trifecta (p=0.01). The preoperative plan of arterial clamping was recorded as clampless, main artery and selective in 22 (24.2%), 22 (24.2%) and 47 (51.6%) cases in Study group vs. 31 (36.9%), 46 (54.8%) and 7 (8.3%) cases in Control group, respectively (p<0.001). At multivariate logistic regressions, the use of 3D model was found to be independent predictor of both selective or super-selective clamping and Trifecta’s achievement.

**Conclusion:**

3D-guided approach to PN increase the adoption of selective clamping and better predict the achievement of Trifecta.

## Introduction

Due to the effect of stage migration (1), increasing proportion of patients with renal tumour are diagnosed with T1 stage disease. Thus, partial nephrectomy (PN) is increasingly adopted as preferred treatment (2–4). Recently, the increasing experience with the robotic approach lead to expand the adoption of robotic PN even in complex T1 (5) and T2 renal mass (6–8). Moreover, the risk of unsuccessful PN with conversion to radical nephrectomy (RN) is higher in challenging cases (9, 10). Thus, a critical and detailed comprehension of tumour’s complexity is essential to achieve optimal success of PN. Nowadays, 3D models facilitate the understanding of renal anatomy (11, 12) and are more accurate to assess with higher accuracy surgical complexity of renal masses compared to 2D imaging (13, 14) and to predict surgical outcomes (15). Likewise in prostate cancer robotic surgery (16–19), 3D models may have strong implications for surgical planning.

Thus, the high-fidelity 3D reconstruction of renal vasculature allows to increase selective clamping (11–13, 20, 21) with potential improvement of functional outcomes. Thus, oncologic and functional outcomes of PN are dependent on quality of tumour resection, renal ischemia and quality and quantity of preserved renal parenchyma (22). Indeed, ideal outcomes of PN should comprehend maximal renal functional preservation, negative surgical margins and no complications: the simultaneous achievement of all three goals has been defined as Trifecta outcomes (23).

The aim of our study was to evaluate the impact of 3D virtual model for a comprehensive assessment of surgical planning and the improvement of the quality of PN.

## Materials and methods

### Population

We prospectively enrolled 195 consecutive patients with clinical diagnoses of single T1-T2 renal mass, scheduled for open, laparoscopic or robot assisted PN by experienced surgeons in each surgical technique at the end of the learning curve at our institution between December 2018 and August 2021. Before surgery, each patient was investigated with high quality chest and abdominal contrast-enhanced CT (slice thickness: 1.25 ÷ 2.5 mm, step interval: 0.8÷ 2.0 mm). Participants signed a written informed consent document. Patients with multiple synchronous renal tumours and with solitary kidney were excluded.The study was approved by our Institutional Ethics Committee (IRB approval 3386/2018).

**Figure 1:**
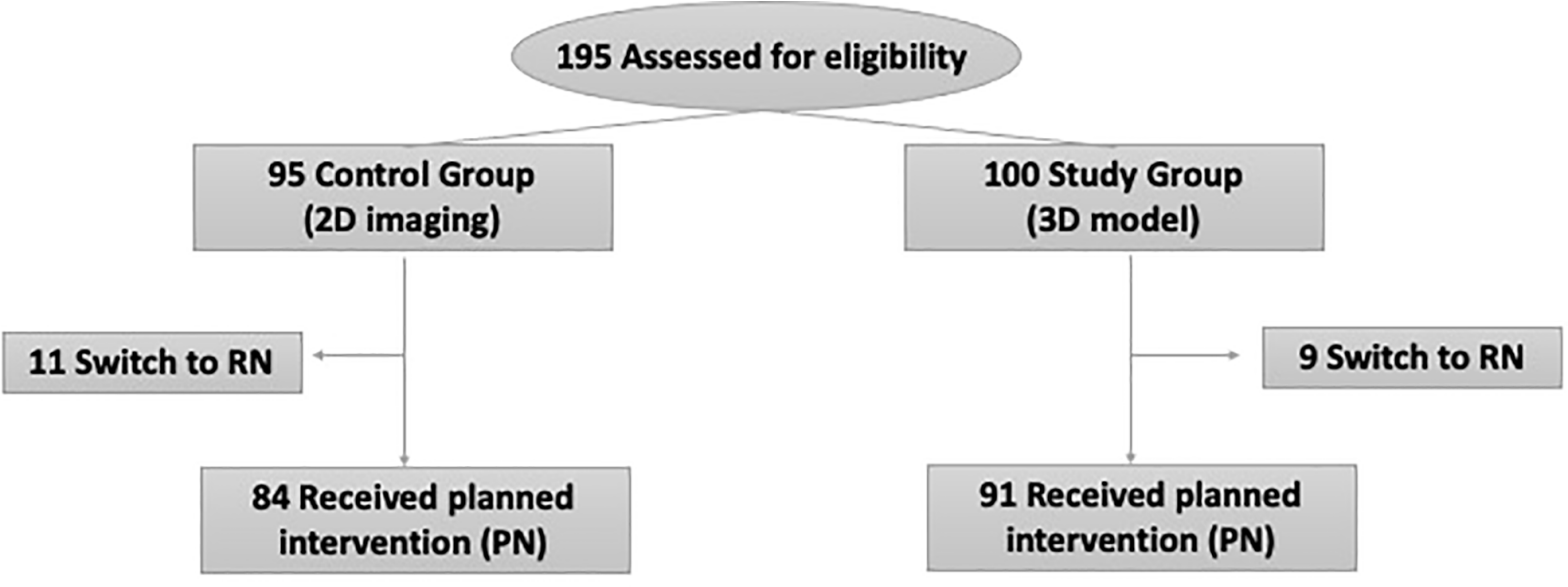
Flow chart of participants in the study.

To evaluate the impact of 3D virtual model on surgical planning and outcomes of PN, patients were stratified in two groups: Study Group (n= 100), including patients scheduled for PN in which the surgeon reviewed both the 2D CT imaging and the 3D virtual model before and during surgery; Control group (n= 95), including patients scheduled for PN in which the surgeon reviewed only the 2D CT imaging before surgery. Overall, 10 individuals referred to PN were switched to radical nephrectomy (RN) before surgery and 10 patients were switched to RN during surgery, thus the final population of patient underwent PN consisted of 175 patients (91 in Study group and 84 in Control group; **Figure 1**).

### 3D modeling

In Study group, all 3D virtual models, based on preoperative high-quality CT scan, were carried out by engineers at eDIMES Lab of the University of Bologna, located at IRCCS, Azienda Ospedaliero-Universitaria, S.Orsola-Malpighi Hospital, as previously described (11, 12).

Briefly, multiple imaging series with different contrast levels were used for the selective identification of each anatomical structure of interest (healthy parenchyma, tumour lesion, extra and intra-renal arterial and venous branches and urinary collecting system [UCS]) in the image segmentation process. Segmentation was achieved using D2P™ software (‘DICOM to PRINT’; 3D Systems Inc., Rock Hill, SC). The segmentation results and the anatomical correctness of the reconstructed 3D virtual models (14, 15) were reviewed and validated by surgeons and radiologists.

For each case in Study group, the surgeon viewed the 3D model before the intervention. Intraoperative viewing of the 3D models was performed at the surgeon’s discretion with the help of an assistant. In some cases, the creation of 3D model was not possible due to organisational reasons, since the reconstruction of 3D model required on average about 3 hours by the bioengineers who are involved also in 3D modelling for different surgical specialities. For these cases, patients underwent surgery with 2D imaging only and were included in the Control group.

### Surgical technique

PNs were performed with open, laparoscopic or robot-assisted approach by three dedicated surgeons with high experience in each surgical approach. The choice of surgical technique was left to the surgeon preference. Open PN was performed through a retroperitoneal flank incision between the XI and XII ribs, as previously described (24). Laparoscopic PN and robot-assisted PN were performed with transperitoneal approach as previously described (25, 26). Laparoscopic PN was performed using three 12 mm trocars and one 5 mm trocar. Robot-assisted PN was performed using the DaVinci^®^ Xi™ Surgical System (Intuitive Surgical Inc., Sunnyvale, CA, USA) in a four-arm configuration with the integrated Firefly™ fluorescence-imaging mode (6). In case of clamping approach to the renal hilum we adopted warm ischemia: a selective (first branch) or super-selective (secondary and tertiary branches) clamping approach was preferred over non selective clamping whenever feasible according to preoperative imaging and intraoperative patients-specific surgical anatomy.

### Surgical planning

The preoperative surgical planning including the need of conversion to RN, the presumed type of arterial clamping technique and the need of UCS suture were evaluated by surgeons on 2D imaging in Control group and both on 2D imaging and 3D virtual models in Study group, consecutively. During surgery, the intraoperative conversion to RN, the effective intraoperative type of clamping and need of UCS suture were recorded and compared to the preoperative planning in both groups.

### Covariates

Demographic and clinical parameters were available for each patient, including age, body mass index, estimated glomerular filtration rate (eGFR; ml/min/1.73 m^2^) and comorbidities classified according to American Society of Anaesthesiologists (ASA) score. The surgical complexity of the renal masses was scored according to PADUA (27) and R.E.N.A.L (28). score based on 2D imaging in both groups. Prospective data were collected: global surgical time, intraoperative estimated blood loss (EBL), conversion to RN, type of arterial clamping, the need of UCS suture, histotype, pathologic stage and grade and positive surgical margin (PSM). The first postoperative eGFR was considered eGFR at discharge. The variation in eGFR from baseline at discharge was estimated for evaluating the impact of the surgical procedure on renal function. Complications within 30 d after surgery were recorded and graded according to the Clavien-Dindo classification (29). Major complications were categorized as Clavien grade III or higher according to European Association of Urology guidelines (30). During follow up, oncologic outcomes (disease recurrence) evaluated by conventional imaging and functional outcomes were recorded.

### Outcomes

The primary outcome of the study was to determinate the impact of 3D virtual model to achieve the Trifecta for PN (defined as the contemporary absence of PSM, major complications and ≤30% postoperative eGFR reduction) (31).

The secondary outcomes were the impact of 3D models in the preoperative planning of PN with regards of rate of conversion to RN, type of arterial clamping and need of UCS suture and the success rate of the preoperative planning concerning the rate of conversion to RN, type of arterial clamping and need of UCS suture.

### Statistical analyses

Chi-squared test, T-student test and Mann-Whitney U-test were used to compare proportion, means and medians, respectively. Statistical analyses consist of several steps. First, in the overall population of patients scheduled for PN, the effective rates of conversion to RN planned before surgery or unanticipated and performed during surgery was compared between Study and Control groups. Second, considering patients who effectively underwent PN, the preoperative planning concerning the type of arterial clamping and the need of UCS suture, the effective intraoperative type of arterial clamping and UCS and the success rate of preoperative planning were compared between Study and Control groups. Third, the Trifecta rate and the causes of Trifecta failure were analyzed between the two groups. Finally, multivariate logistic regressions were used to identify independent predictors of selective or super-selective clamping and of Trifecta’s achievement, basing on significant predictors at univariate analyses. Covariates consist of follows: age, ASA score, use of 3D models, surgical technique (open, laparoscopic or robotic), PADUA score (Model 1), RENAL score (Model 2). Multivariate logistic regression models to predict conversion to RN and UCS suture were not employed due to limited number of events.

A p value of <0.05 was considered statistically significant. All statistical tests were performed using SPSS 22.0 for Windows.

## Results

**Figure 2:**
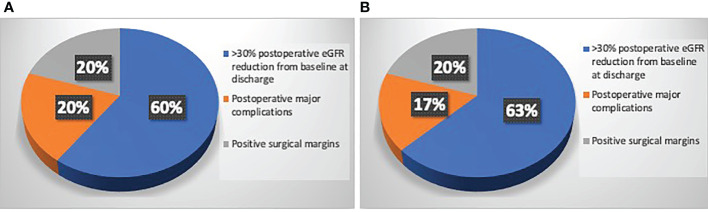
**(A)** Causes of Trifecta’s failure in patients underwent PN in Study group who did not reach the Trifecta (n=18/91); **(B)** Causes of Trifecta’s failure in patients underwent PN in Control group who did not reach the Trifecta (n=31/84).

**Table 1** depicts baseline characteristics in the overall population of patients scheduled for PN (n=195). Surgery was performed with open, laparoscopic or robotic approach in 37 (19%), 25 (12.8%) and 133 (68.2%) cases, respectively. The conversion to RN was planned before surgery in 77.8% and in 27.3% of cases in Study group after revision of the 3D model and in Control group after revision of 2D imaging, respectively (p=0.03). Considering the subgroup of patients referred to PN (n=175), the preoperative plan of arterial clamping was recorded as clampless, main artery, selective or super-selective in 22 (24.2%), 22 (24.2%), and 47 (51.6%) cases in Study group vs. 31 (36.9%), 46 (54.8%) and 7 (8.3%) cases in Control group, respectively (p<0.001). During surgery, the intraoperative management of the renal pedicle was done as preoperatively planned in 63.6% vs. 74.5% of cases for clampless approach (p=0.05), in 90.1% vs. 76% of cases for main artery clamping (p=0.005) and in 61.7% vs. 28.6% of cases for selective and super-selective clamping (p<0.001) in Study group vs. Control group, respectively. The effective intraoperative need of UCS suture was done as preoperatively planned in 66.6% and 26.1% in Study and Control group, respectively (p=0.004; **Table 2**). **Table 3** shows intra, perioperative, pathologic and postoperative characteristics of individuals who effectively underwent PN (n=175). Overall, 11 and 6 patients in Study group vs. 13 and 4 individuals in Control group experienced Clavien grade I-II and Clavien grade III postoperative complications, respectively (p=0.5). Types of intra- and postoperative complications are reported in **Table 4**. At mean follow up of 12.5 months, patients underwent PN procedure experienced a mean decrease of eGFR value of -5.8% at discharge. Overall, 80.6% of patients in Study group and 73.4% in Control group had ≤30% postoperative eGFR reduction from baseline at discharge (p=0.01). Overall, 73 (80.2%) and 53 (63.1%) patients achieved the Trifecta for PN in Study and Control group, respectively (p=0.01). **Figure 2** depicts the causes of Trifecta’s failure in the two groups. At multivariate logistic regressions, the use of 3D model was found to be independent predictor of both adoption of selective or super-selective clamping (Odd Radio [OR]:5.26 in model 1 and OR:5.04 in model 2; all p ≤ 0.001) and of Trifecta’s achievement (OR:2.42 in model 1 and OR:2.41 in model 2; all p ≤ 0.02; **Table 5**).

**Table 1:** Patient characteristics and descriptive statistics in the overall population (n=195).

	Control Group (2D imaging)	Study Group (3D model)	P value
No. of patients, n (%)	95	100	
Age (years)			0.7
Median (IQR)	66 (54-72)	65 (58-70)	
Gender, n (%)			
Male	64 (69.6)	68 (68)	0.8
Female	28 (30.4)	32 (32)
BMI (Kg/m^2^)			
Median (IQR)	26.2 (23.9-29)	26.4 (24-29.5)	0.8
ASA score, n (%)			
1-2	57 (60)	71 (71.7)	0.9
3-4	38 (40)	28 (28.3)
Pre-operative Hb (g/dl)			
Median (IQR)	14.3 (13-15.3)	14.4 (13.6-15-4)	0.7
Pre-operative serum Creatinine (mg/dl)			
Median (IQR)	0.88 (0.72-1.01)	0.88 (0.78-0.98)	0.6
Pre-operative eGFR (ml/min)			
Median (IQR)	87 (74-98)	89 (74-98)	0.7
Clinical stage, n (%)			0.9
cT1a	69 (72.6)	75 (75)
cT1b	23 (24.2)	23 (23)
cT2a	3 (3.2)	2 (2)
PADUA score, n (%)			
Median (IQR)	8 (7-10)	8 (7-10)	0.6
PADUA risk, n (%)			
Low	30 (31.6)	33 (33)	0.9
Intermediate	39 (41.1)	40 (40)
High	26 (27.4)	27 (27)
RENAL score, n (%)			
Median (IQR)	7 (6-9)	7 (6-9)	0.6
RENAL risk, n (%)			
Low	33 (34.7)	38 (38)	0.9
Intermediate	50 (52.6)	49 (49)
High	12 (12.6)	13 (13)
Clinical lesion diameter at CT scan (cm)			
Median (IQR)	3.2 (2.2-4.2)	3 (2.2-4)	0.9
Surgical technique, n (%)			
Open	20 (21.1)	17 (17)	0.2
Laparoscopic	16 (16.8)	9 (9)
Robotic	59 (62.1)	74 (74)
Conversion to RN n (%)	11 (11.6)	9 (9)	0.6
Conversion to RN*, n (%)			
Pre-planned (before surgery)	3 (27.3)	7 (77.8)	0.03
Not planned (during surgery)	8 (72.7)	2 (22.2)

3D, 3 Dimensional; IQR, Interquartile Range; ASA, American Society of Anesthesiologists; BMI, Body Mass Index; Hb, hemoglobin; eGFR, estimated glomerular filtration rate; CT, computed tomography.

*Considering patient with conversion to radical nephrectomy.

**Table 2:** Sub-analysis in patients underwent PN (n= 175) to compare the preoperative planning and the intraoperative approach to the renal hilum and caliceal system suturing.

	Control Group (2D imaging)	Study Group (3D model)	P value
Preoperative planning of arterial clamping, n (%)			< 0.001
Clampless	31 (36.9)	22 (24.2)	
Main artery	46 (54.8)	22 (24.2)	
Selective (I order branch) or Super selective (II – III order branch)	7 (8.3)	47 (51.6)	
Effective Intraoperative arterial clamping, n (%)			< 0.001
Clampless	31 (36.9)	26 (28.6)
Main artery	45 (53.6)	32 (35.2)
Selective (I order branch) or Super selective (II – III order branch)	8 (9.5)	33 (36.3)
Effective Intraoperative Clamping approach as previously planned, n (%)			
Clampless	23 (74.2)	14 (63.6)	0.05
Main artery	35 (76.1)	20 (90.1)	0.005
Selective (I order branch) or Super selective (II – III order branch)	2 (28.6)	29 (61.7)	<0.001
Preoperative planning of caliceal suture, n (%)	23 (27.7)	27 (29.7)	0.8
Intraoperative need of caliceal suture, n (%)	7 (8.3)	21 (23.3)	0.007
Effective Intraoperative need of caliceal suture as previously planned, n (%)	6 (26.1)	18 (66.6)	0.004

**Table 3:** Intraoperative, peri-operative, pathologic and postoperative characteristics in the overall population underwent PN (n=175).

	Control Group (2D imaging)	Study Group (3D model)	P value
WIT (min)*			0.9
Median (IQR)	14 (10 -18)	13.5 (10 -20)	
Operative time (min)			0.2
Mean ± SD	164± 64	178 ± 63
Resection technique, n (%)			0.08
Standard partial nephrectomy	32 (38.1)	23 (25.6)
Simple enucleation	52 (61.9)	67 (74.4)
Time of renal defatting (min)			0.9
Mean ± SD	23 ± 19	23 ± 13
Time of hilum dissection (min)			0.9
Mean ± SD	19 ± 13	19 ± 11
Time of enucleation (min)			0.9
Mean ± SD	11 ± 7	12 ± 15
Estimated blood loss (ml)			0.6
Mean ± SD	140 ± 50	130 ± 40
Intraoperative complications, n (%)	5 (6)	4 (4.4)	0.6
Post-operative complications grade, n (%)			0.8
Overall	17 (20.2)	17 (18.7)
-Clavien 1-2	13 (15.5)	11 (12.1)
-Clavien 3	4 (4.8)	6 (6.6)
Positive Surgical Margins, n (%)	7 (8.3)	4 (4.4)	0.3
Length of stay (days)			0.6
Median (IQR)	4 (4-5)	4 (3-5)
Pathological lesion diameter (cm)			0.6
Mean ± SD	3 ± 1.5	3.4 ± 1.5
Pathology, n (%)			0.8
Benign	18 (21.4)	17 (18.7)
Clear cell carcinoma	38 (45.2)	47 (51.6)
Papillary carcinoma	15 (17.9)	13 (14.3)
Chromophobe carcinoma	9 (10.7)	12 (13.2)
Other malignancies	4 (4.8)	2 (2.2)
Pathological stage, n (%)			0.3
pT1a	59 (70.2)	65 (71.4)
pT1b	17 (20.2)	23 (25.3)
pT2a	1 (2.2)	1 (1.1)
pT3a	7 (8.3)	2 (2.2)
Follow up time, months			
Mean ± SD	15 ± 12	13 ± 11	0.06
Postoperative eGFR (ml/min/1.73 m^2^) at discharge, Mean ± SD	73 ± 26	77 ± 21	0.2
Variation in eGFR (ml/min) from baseline at discharge (%)			
Mean ± SD	-6.2 ± 20	-5.5 ± 20	0.2
≤30% postoperative eGFR reduction from baseline at discharge, n (%)	62 (73.4)	79 (80.6)	0.03
Recurrence, n (%)			
Yes	0 (0)	2 (3.1)	0.4
Trifecta achievement, n (%)	53 (63.1)	73 (80.2)	0.01

*Considering patient underwent on clamping approach; WIT, Warm Ischemia Time; SD, standard deviation; eGFR, estimated glomerular filtration rate.

Trifecta achievement: contemporary absence of positive surgical margins, major complications and ≤30% postoperative eGFR reduction.

**Table 4:** Intraoperative and postoperative complications in the overall population (n=195).

	Control Group (2D model)	Study Group (3D imaging)	P value
INTRAOPERATIVE COMPLICATIONS			
Bleeding, n (%)	4 (4.2)	3 (3)	0.7
Pleuric lesion, n (%)	1 (1.1)	1 (1)	0.9
POSTOPERATIVE COMPLICATIONS (<30 days)			
TVP-TEP, n (%)	0 (0)	1 (1)	0.3
Cardiac, n (%)	4 (4.2)	4 (4)	0.9
Ileus, n (%)	0 (0)	1 (1)	0.3
Infection, n (%)	7 (7.4)	5 (5)	0.5
AKI, n (%)	4 (95)	5 (5)	0.8
Bleeding, n (%)	6 (6.3)	5 (5)	0.7
Pleural effusion, n (%)	1 (1.1)	0 (0)	0.3
Pneumothorax, n (%)	1 (1.1)	0 (0)	0.3
Urinary leakage, n (%)	2 (2.1)	1 (1)	0.5

**Table 5:** Multivariate logistic regression analyses to predict the adoption of selective or super-selective clamping and the achievement of Trifecta in patients underwent PN (175), evaluating separately PADUA score (Model 1) and R.E.N.A.L. score (Model 2).

	ADOPTION OF SELECTIVE OR SUPER-SELECTIVE CLAMPING				ACHIEVEMENT OF TRIFECTA			
	MODEL 1		MODEL 2		MODEL 1		MODEL 2	
	OR (95% CI)	P value	OR (95% CI)	P value	OR (95% CI)	P value	OR (95% CI)	P value
Age at surgery (years)	–	–	–	–	0.99 (0.95-1.02)	0.8	0.98 (0.95-1.02)	0.9
ASA score	–	–	–	–				
1-2					1.0 (Ref)	0.3	1.0 (Ref)	0.4
≥3					1.51 (0.64-3.72)		1.47 (0.62-3.50)	
PADUA score	1.19 (0.94-1.49)	0.1	–	–	0.86 (0.70-1.07)	0.2	–	–
RENAL score	–	–	1.07 (0.86-1.32)	0.6	–	–	0.86 (0.71-1.04)	0.1
3D virtual model								
No	1.0 (Ref)	<0.001	1.0 (Ref)	<0.001	1.0 (Ref)	0.01	1.0 (Ref)	0.02
Yes	5.26 (2.22-12.50)		5.04 (2.14-11.88)		2.42 (1.19-4.92)		2.41 (1.18-4.90)	
Surgical approach								
Open Laparoscopic Robot assisted	1.0 (Ref) 0.33 (0.03-3.31) 1.68 (0.55-5.14)	0.2 0.3 0.4	1.0 (Ref) 0.28 (0.03-2.83) 1.60 (0.53-4.86)	0.2 0.3 0.4	1.0 (Ref) 0.59 (0.15-2.35) 0.85 (0.28-2.49)	0.7 0.5 0.8	1.0 (Ref) 0.59 (.015-2.32) 0.83 (0.28-2.43)	0.4 0.7 0.1

OR, Odd Ratio; CI, Confidence Interval; ASA, American Society of Anesthesiologists.

## Discussion

In recent years several technologic improvements have been introduced with the aim to increase the quality of renal surgery. The main goal of PN includes complete tumour excision with negative surgical margins, as well as reduced complications and damage to the healthy renal parenchyma as effect of resection of peritumoral renal tissue or ischemic damage (i.e. Trifecta). Indeed, to reduce the ischemic damage during PN, a non-global ischemia techniques have been proposed (32), despite the effect of selective clamping on renal function impairment is still debated. However, the adoption of selective clamping remained less popular in the pre-robotic era due to the need of precise dissection of segmental arterial branches. With the advent of robotics, a more precise surgery that allowed meticulous dissection of higher-order renal arteries was made possible (32). Nevertheless, PN is a complex surgical intervention and an accurate presurgical planning is the key for a good quality of PN outcomes. Before surgery, many aspects should be investigated: planning a conversion to RN, surgical technique, transperitoneal or retroperitoneal approach, selection of blood vessels for clamping, tumor resection margin and need of UCS suture. In the current era of precision surgery, the introduction of 3D models allow to simplify the anatomical knowledge of renal mass and to easily assess the surgical complexity (14), allowing a patient-tailored approach for PN (15).

**Figure 3:**
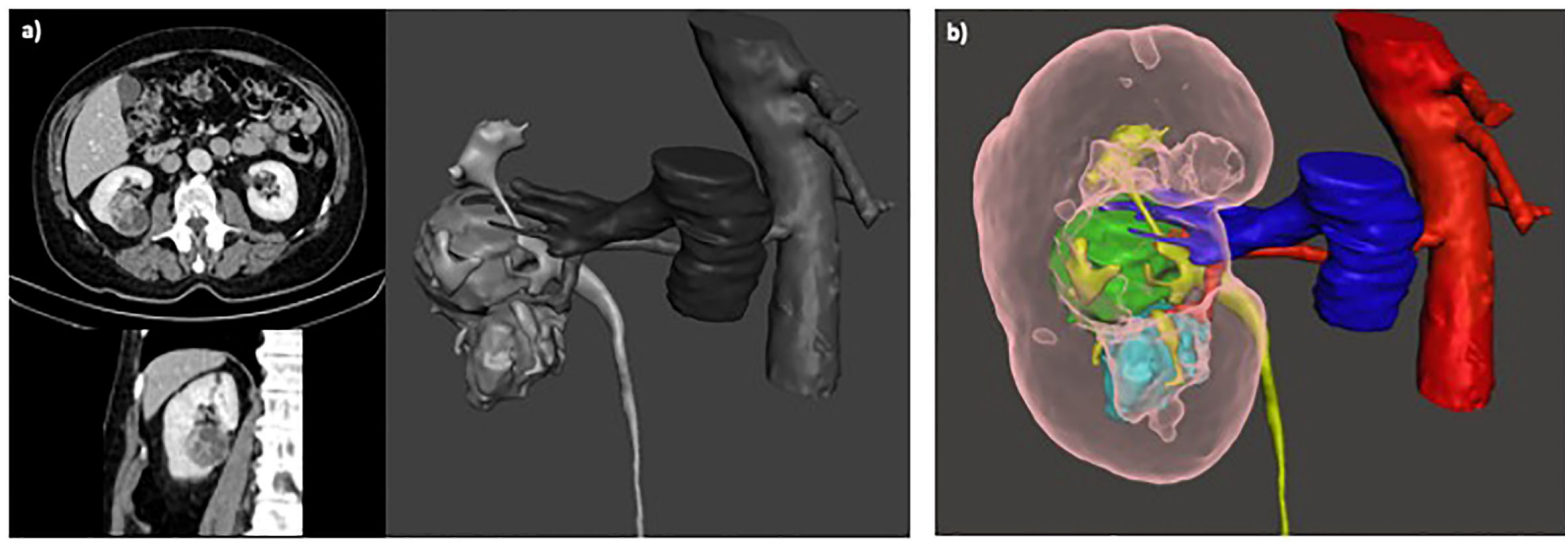
**(A)** Surgical planning of scheduled PN based on 2D imaging; **(B)** After revision of 3D model the planning of surgery was converted to RN before surgery, due to suspicious invasion of urinary collecting system and renal sinus.

**Figure 4:**
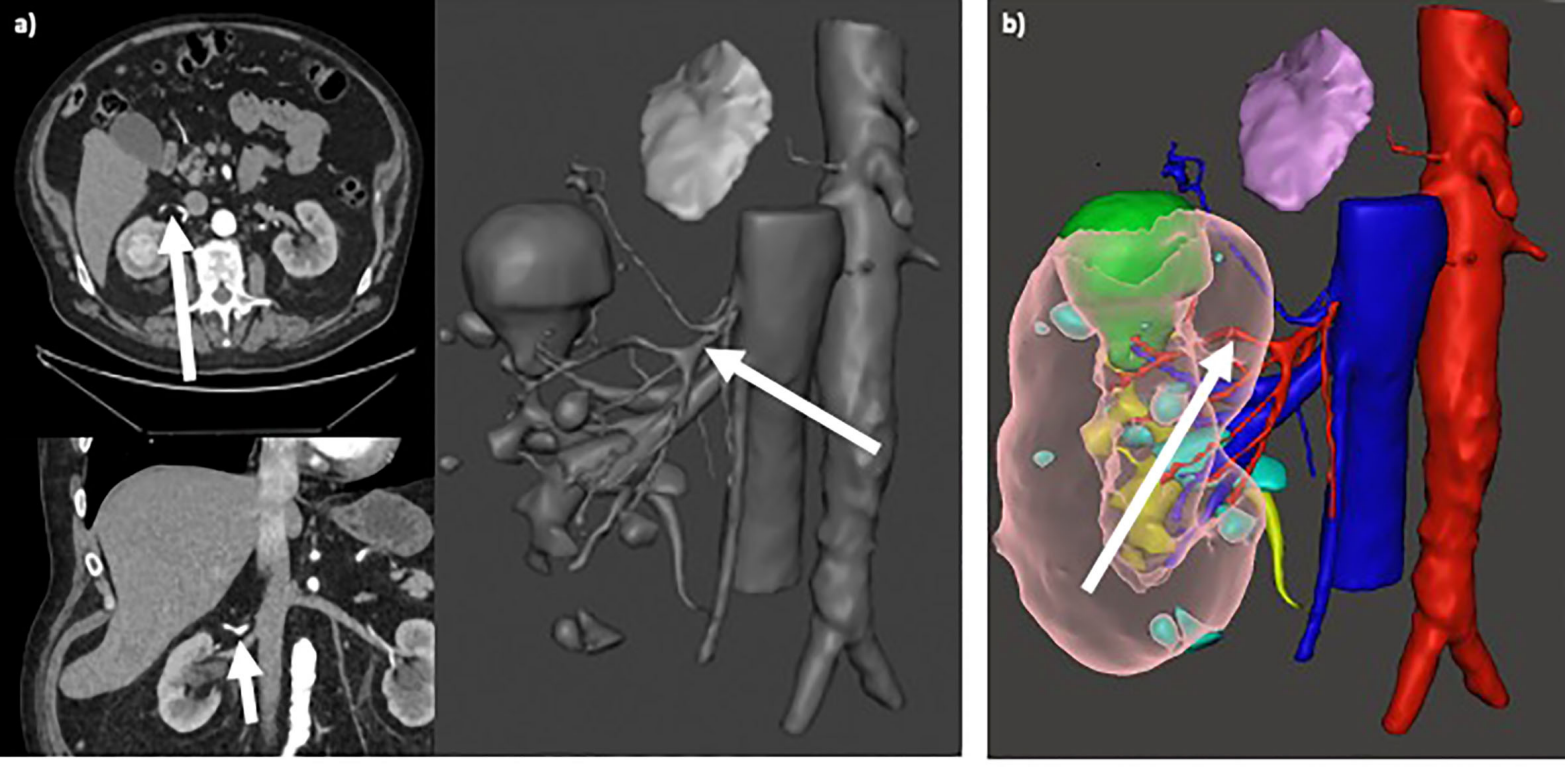
**(A)** Selective clamping of primary arterial branching (arrows in axial/coronal views and in 3D rendering), resulting from preoperative planning based on standard 2D CT imaging; **(B)** Super-selective clamping of tertiary arterial branching (arrow), resulting from preoperative planning based on 3D model.

Several points of our study are remarkable. First, the use of 3D model allows to significantly reduce the intraoperative conversions to RN. In our cohort the overall rate of conversion to RN (10.3%) is consistent with previously reported data from very high-volume centres in which it ranges from 3.1% (10) including only robotic cases to 12.4% (33) including both open, laparoscopic and robotic approach. Reason for unsuccessful PN may consist of patients-related factors, tumor-related features (9, 34) or surgeons and center experience (3, 33). As consequence, a precise evaluation of complexity of renal mass is the key to achieve successful PN. Our results suggest that the use of 3D model may predict the occurrence of unsuccessful PN, changing the indication from PN to RN before surgery allowing a better patients selection for PN, reducing the intraoperative conversion to RN that may be associated with longer operating times, increased blood loss, and worse postoperative renal function compared to non-converted RN (9, 10) (**Figure 3**). Second, our data confirmed that the effective intraoperative adoption of selective clamping was significantly higher in patients with 3D model available (36.3%) compared to patients with only 2D imaging (9.5%; p<0,001). Contrarily to previous reports by Michiels et al. (22) in which most patients with 3D model underwent PN without clamping (50.9%), while the vast majority of patients without 3D model underwent PN with main artery clamping (91.7%), in our cohort the rate of effective clampless approach was significantly higher in control group (36.9%) compared to patients with 3D model (28.6%). This could be due to surgeons’ and centres’ experience and preference: in our experience we noticed that when 3D model is not available the surgeon is more prone to clamp the main artery in case of complex mass or to perform clampless PN in case of easier cases. Besides, when the 3D model is available, a selective or super-selective clamping is planned and attempt even in easier cases that would have been treated with clampless approach whenever the 3D model was not available, to achieve better bleeding control. To note, the protective effect of 3D models on postoperative renal function may be due to higher adoption of selective clamping (36.3%) in our cohort and higher adoption of clampless approach (50.9%) in the series by Michiels et al (22). Moreover, the potential benefit of higher adoption of clampless approach (36.9%) in control group on postoperative renal function may be mitigated by significant adoption of main artery clamping (53.6%). Of note, the preoperative planning using the 3D virtual model is more accurate and surgeons revealed higher adherence to the preoperative planning during surgery, with lower risk of unexpected events or to change the predetermined plan of surgery (**Figure 4**).

Third, the use of 3D models led to improve quality of PN since the overall Trifecta achievement was significantly higher in study group (80.2%) compared to control group (63.1%). Similarly to other experience (22), in our cohort, the use of 3D model is independent predictor of Trifecta achievement even adjusting for surgical technique (6). To note, these findings may be limited by selection bias due to lacking of randomizations between the two groups, since patients in study group had higher use of robotic approach, better comorbidities profile and lower tumour’s volume compared to control group, despite no significant difference at baseline characteristics. Finally, the proportion of patients with ≤30% postoperative eGFR reduction from baseline at discharge was significantly higher in Study group. Thus, the higher adoption of selective clamping provided by 3D models may reduce the level of nephron loss due to hypoxia (32), however, long terms data are needed to assess the real effect of 3D-guided PN on renal functional outcomes.

Despite several strengths, our study is not avoided from limitations. First, the lack of randomization between the two groups. Second, the inclusion of patients who underwent surgery with open, laparoscopic and robotic approach may affect surgical outcomes. Third, 3D model reconstruction is intrinsically affected by inaccuracies due to errors that may occur for poor CT scan resolution and/or inaccurate image segmentation process. Finally, limited follow up did not allow to assess conclusion concerning the real impact of 3D-guided PN on long term residual renal function compared to standard 2D approach.

Despite such limitations, the technologic progress would improve the precision of 3D reconstruction to simplify surgical planning and the intraoperative 3D navigation during PN. In the next future, 3D virtual models may represent an essential tool for multiple needs of NSS: patients’ counseling, trainers’ education, standardize the surgical complexity, improve the efficiency of surgical planning and the quality of patient-tailored surgery.

In conclusion, the use of 3D models may improve the efficiency of surgical planning, reducing the risk of conversion to RN during surgery. Moreover, a 3D-guided approach to PN increase the adoption of selective clamping and better predict the achievement of Trifecta. Thus, the 3D virtual models applied to PN may improve the quality of surgery with potential implication on patient’s outcomes.

## Data availability statement

The raw data supporting the conclusions of this article will be made available by the authors, without undue reservation.

## Ethics statement

Approval of the research protocol by an Institutional Reviewer Board: IRB approval 3386/2018. The patients/participants provided their written informed consent to participate in this study.

## Author contributions

LB, LC, FC, RS contributed to conception and design of the study. FC, GC, CG, AB, LT organized the database. BB, PP, MD, SB performed the statistical analysis. LB, BB, LC, EM, SL wrote the first draft of the manuscript. CG, GC, RG, EB, RS, LT, AB wrote sections of the manuscript. All authors contributed to manuscript revision, read, and approved the submitted version.

## Funding

The work reported in this publication was funded by the Italian Ministry of Health, **RC-2022-2773318.**

## Acknowledgments

Authors acknowledge Fondazione CARISBO of Bologna (Italy).

## Conflict of interest

The authors declare that the research was conducted in the absence of any commercial or financial relationships that could be construed as a potential conflict of interest.

## Publisher’s note

All claims expressed in this article are solely those of the authors and do not necessarily represent those of their affiliated organizations, or those of the publisher, the editors and the reviewers. Any product that may be evaluated in this article, or claim that may be made by its manufacturer, is not guaranteed or endorsed by the publisher.
